# Subcutaneous nodules on the elbows of a teenager

**DOI:** 10.1016/j.jdcr.2026.05.001

**Published:** 2026-05-09

**Authors:** Arman Haveric, Jayci G. Rhein, Jason B. Lee, Shayan Waseh, Sylvia Hsu, Afton R. Metkowski

**Affiliations:** a Department of Dermatology, Temple University Hospital, Philadelphia, Pennsylvania; b Department of Dermatology and Cutaneous Biology, Thomas Jefferson University, Philadelphia, Pennsylvania

**Keywords:** hyperlipidemia, sitosterolemia, xanthoma

## Case presentation

**Fig 1:**
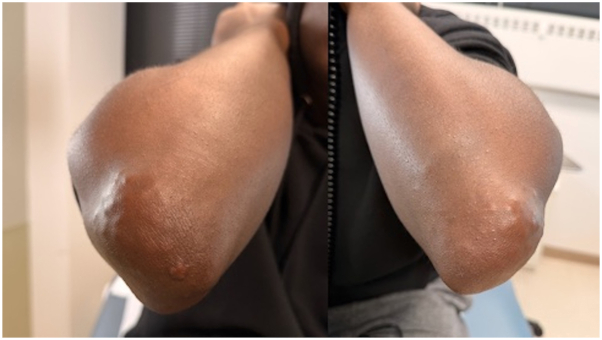
Subcutaneous nodules on bilateral elbows.

A 17-y-old boy with no past medical history presented with a 1-y history of asymptomatic, subcutaneous nodules on the bilateral elbows (Fig 1). Family history was significant for myocardial infarction at age 48 in his maternal aunt (the half-sister of his biological mother).

**Fig 2:**
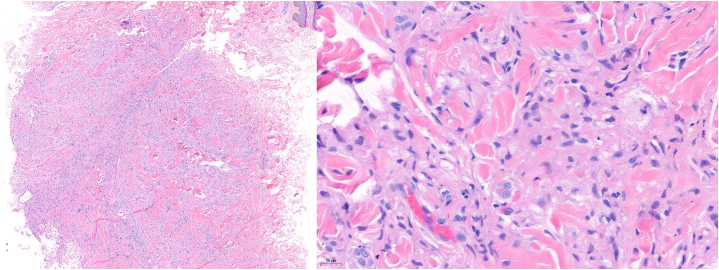
Histopathologic examination demonstrates an interstitial histiocytic infiltrate at scanning (H&E 50×) magnification (*left*) which is revealed to contain lipid-laden foamy cytoplasm (H&E 400×) (*right*).

Two 4-mm punch biopsies were performed, and histopathologic assessment revealed numerous lipid-laden histiocytes in the dermis and subcutis were noted, more consistent with xanthomas (Fig 2). A lipid panel was ordered and revealed marked hypercholesterolemia, hypertriglyceridemia, and elevated low-density lipoprotein cholesterol (Table I).

**Table I:** Lipid profile

Laboratory value	Measured value	Reference range
Cholesterol, total	**351**	100-169 mg/dL
Triglycerides	**94**	0-89 mg/dL
High-density lipoprotein	51	>39 mg/dL
Very low-density lipoprotein cholesterol, calculated	15	5-40 mg/dL
Low-density lipoprotein, calculated	**285**	0-109 mg/dL

Bold values indicate that they are outside the reference range.

The patient was then referred to a pediatric multidisciplinary metabolism clinic. Plasma gas chromatography-mass spectrometry revealed elevated plasma sterol concentrations (Table II). The patient was referred to cardiology for management of long-term cardiovascular risk.

**Table II:** Plasma sterols panel

Laboratory value	Measured value	Reference range
Cholestanol	**38.5**	≤6.0 mg/L
Dihydro-testis meiosis-activating sterol	**0.4**	≤0.3 mg/L
Stigmasterol	**21.9**	≤0.5 mg/L

Bold values indicate that they are outside the reference range.

## Question: Which is the most appropriate first-line medical therapy for this patient?

**A.** Atorvastatin
**B.** PCSK9 inhibitor (eg, evolocumab)
**C.** Gemfibrozil
**D.** Ezetimibe
**E.** Intralesional triamcinolone injections

Correct answer: **D.** Ezetimibe.

## Discussion

Sitosterolemia is an autosomal recessive lipid disorder characterized by elevated plasma concentrations of plant sterols.^1^ In sitosterolemia, biallelic loss-of-function mutations in *ABCG5* or *ABCG8* genes increase intestinal absorption and impair hepatic excretion of plant sterols, including sitosterol, campesterol, and stigmasterol, leading to their gradual accumulation in serum and tissues.^2^

Distinguishing sitosterolemia from the more common entity familial hypercholesterolemia (FH) is challenging, as clinical, histologic, and routine laboratory features can be identical, including tendon xanthomas, elevated low-density lipoprotein cholesterol, and premature atherosclerosis.^2^^,^^3^ Differentiation is crucial, however, as treatment for sitosterolemia is unique.

Tuberous and tendinous xanthomas may be the first presenting sign of sitosterolemia, most typically occurring on the heels, knees, elbows, and buttocks and the Achilles or hand tendons.^1^^,^^3^ Periorbital xanthelasma is more typical in FH,^4^ but has also been reported in adults with sitosterolemia.^2^ Intertriginous (particularly interdigital) planar xanthomas are more characteristic of homozygous FH,^4^ although one case report has documented planar xanthomas in the flexural creases of an infant with sitosterolemia.^1^ Xanthomas in sitosterolemia tend to manifest prepubertally and sometimes in infancy,^1^^,^^5^ whereas in heterozygous FH, they typically appear in adulthood, and in homozygous FH during childhood.^4^^,^^5^ Unfortunately, no single physical examination finding is pathognomonic for sitosterolemia. Instead, diagnosis is predicated on distinct laboratory findings.

In up to 35% of cases, sitosterolemia is characterized by hematologic derangements, including hemolytic anemia, stomatocytosis, or macrothrombocytopenia,^5^ features not seen in FH.^1^^,^^3^^,^^4^ Additionally, because sitosterolemia results from hyperabsorption of dietary sterols rather than increased endogenous cholesterol synthesis, statins are not therapeutic.^1^ Thus, a poor response to statin therapy in a patient with xanthomas and hypercholesterolemia should prompt evaluation for sitosterolemia. Finally, because it is autosomal recessive, sitosterolemia should be suspected in patients with xanthomas with no family history of hyperlipidemia or premature atherosclerosis. Diagnosis is confirmed through gas or liquid chromatography, demonstrating elevated plant sterol concentrations, and/or genetic testing.^1^^,^^3^

Ezetimibe, which inhibits Niemann-Pick C1-like 1-mediated sterol uptake, is the first-line medical therapy.^3^ Combination therapy with ezetimibe and dietary restriction of plant sterols (including vegetable oils, margarine, legumes, chocolate, avocado, and soy) has been shown to drastically improve lipid and plasma phytosterol levels and frequently leads to xanthoma regression.^1^ Bile acid resins, such as cholestyramine, may be used adjunctively.^1^ Dermatologists may play a crucial and possibly lifesaving role by identifying sitosterolemia, as early management is required to avoid significant cardiovascular morbidity.^1^^,^^3^

## Conflicts of interest

None disclosed.

## References

[1] Myrie S.B., Steiner R.D., Mymin D., Adam M.P., Bick S., Mirzaa G.M. (2013). Genereviews® [Internet].

[2] Tada H., Nohara A., Inazu A., Sakuma N., Mabuchi H., Kawashiri M.A. (2018). Sitosterolemia, hypercholesterolemia, and coronary artery disease. J Atheroscler Thromb.

[3] Rocha V.Z., Tada M.T., Chacra A.P.M. (2023). Update on sitosterolemia and atherosclerosis. Curr Atheroscler Rep.

[4] Xia Y., Duan Y., Zheng W. (2022). Clinical, genetic profile and therapy evaluation of 55 children and 5 adults with sitosterolemia. J Clin Lipidol.

[5] Tada M.T., Rocha V.Z., Lima I.R. (2022). Screening of ABCG5 and ABCG8 genes for sitosterolemia in a familial hypercholesterolemia cascade screening program. Circ Genom Precis Med.

